# Deciphering the Causal Relationship between Sodium-glucose Cotransporter 2 Inhibition and Cancer Risks: A Comprehensive Mendelian Randomization Study

**DOI:** 10.7150/jca.96435

**Published:** 2024-05-28

**Authors:** Liyin Zhang, Baorui Xue, Fangyang Yu, Yao Yin, Si Jin

**Affiliations:** Department of Endocrinology, Institute of Geriatric Medicine, Liyuan Hospital, Tongji Medical College, Huazhong University of Science and Technology, 39 Lake Road, East Lake Ecological Scenic, Wuhan 430077, Hubei, China.

**Keywords:** Mendelian randomization, sodium-glucose cotransporter 2 inhibition, cancer, Genetics

## Abstract

**Background:** Controversy persists regarding the effects of sodium-glucose cotransporter 2 (SGLT2) inhibitors on cancer. The underlying causal relationship remains unclear.

**Method:** A two-sample Mendelian randomization (MR) strategy was employed to investigate the causal associations between SGLT2 inhibitors and 26 site-specific malignancies. Instrumental variants strongly associated with SLC5A2 gene expression and glycated hemoglobin A1c levels were identified as the genetic proxy for SGLT2 inhibition. Cancer-related outcome datasets sourced from the OpenGWAS project were separated into discovery and replication datasets. The meta-analysis was conducted to determine the final causality.

**Results:** Genetically proxied SGLT2 inhibition showed a significant association with bronchial and lung cancer (beta: -0.028 [-0.041, -0.015], P < 0.001), bladder cancer (beta: 0.018 [0.008, 0.027], P < 0.001), prostate cancer (beta: 1.168 [0.594, 1.742], P < 0.001), cervical cancer (beta: -0.019 [-0.031, -0.008], P = 0.001), corpus uterine cancer (beta: 0.015 [0.006, 0.025], P = 0.001) and non-melanoma skin cancer (beta: -0.080 [-0.116, -0.044], P < 0.001) in the discovery cohort. The suggestive causal effect of SGLT2 inhibition on the increased risk of cervical cancer (beta: 3.241 [0.855, 5.627], P = 0.008) and lymphoid leukemia (beta: 4.126 [0.383, 7.868], P = 0.031) was found in the replication cohort. The combined causality of the following types of cancer was observed to remain significant after meta-analysis: bronchial and lung cancer, bladder cancer, prostate cancer, corpus uterine cancer, and non-melanoma skin cancer (all P ≤ 0.001).

**Conclusion:** For the first time we discovered that the SGLT2 inhibition may exert protection on bronchial and lung cancer and non-melanoma skin cancer from a genetic perspective. However, suggestive higher cancer risks of bladder, prostate, and corpus uteri were also noted, which warrants real-world data validation in the future.

## Introduction

Currently, the sodium-glucose cotransporter 2 (SGLT2) inhibitors, which are guidelines-endorsed oral antihyperglycemic drugs for individuals with type 2 diabetes mellitus (T2DM), have garnered attention due to their beneficial impact on cardiorenal outcomes, kidney and cardiovascular function 1-5.

Previous researches have explored the association between SGLT2 inhibitors and different types of cancer 6-8, but no clear conclusions have been drawn regarding a causal relationship. Pharmacological inhibition of SGLT2 has demonstrated a promising prognosis in rodent models of solid malignancies affecting the liver, lung, pancreas, and colon 9-13. Studies have shown that SGLT2 is present in pancreatic and prostate tumors, as well as glioblastomas, and functions as a glucose transporter in cancer cells 11, 14. Additionally, a prior study based on the population suggested that starting treatment with SGLT2 inhibitors led to increased survival rates in patients with hepatocellular carcinoma 15. However, a meta-analysis suggested that there was no connection found between SGLT2 inhibitors and an increased incidence of overall cancer 8, although it was indicated that they might induce tumors in rats 16. Nevertheless, it should be noted that another meta-analysis integrating multiple research findings found that the SGLT2 inhibitor use showed a clear correlation with a decrease in the risk of developing cancer 17. Therefore, a novel comprehensive analysis of SGLT2 inhibition on cancers is urgently needed.

Given conflicting results regarding possible associations with cancers and the intrinsic limitations of traditional observational studies such as those introduced by confounders, our study utilized Mendelian randomization (MR) to clarify the cancer risk associated with SGLT2 inhibitors. MR is a powerful method in the field of causal inference that leverages genetic variants known as single nucleotide polymorphisms as instrumental variables to help establish causation between an exposure and an outcome 18. A previous MR study reported a potential causal relationship between long-term exposure to SGLT2 inhibitors and the increased risk of prostate and bladder cancer 19. Yet, to the best of our knowledge, there have been no MR studies that have thoroughly evaluated the cause-and-effect association between SGLT2 inhibitors and 26 site-specific cancers at the whole-body level.

## Materials and Methods

### Study design

**Figure 1:**
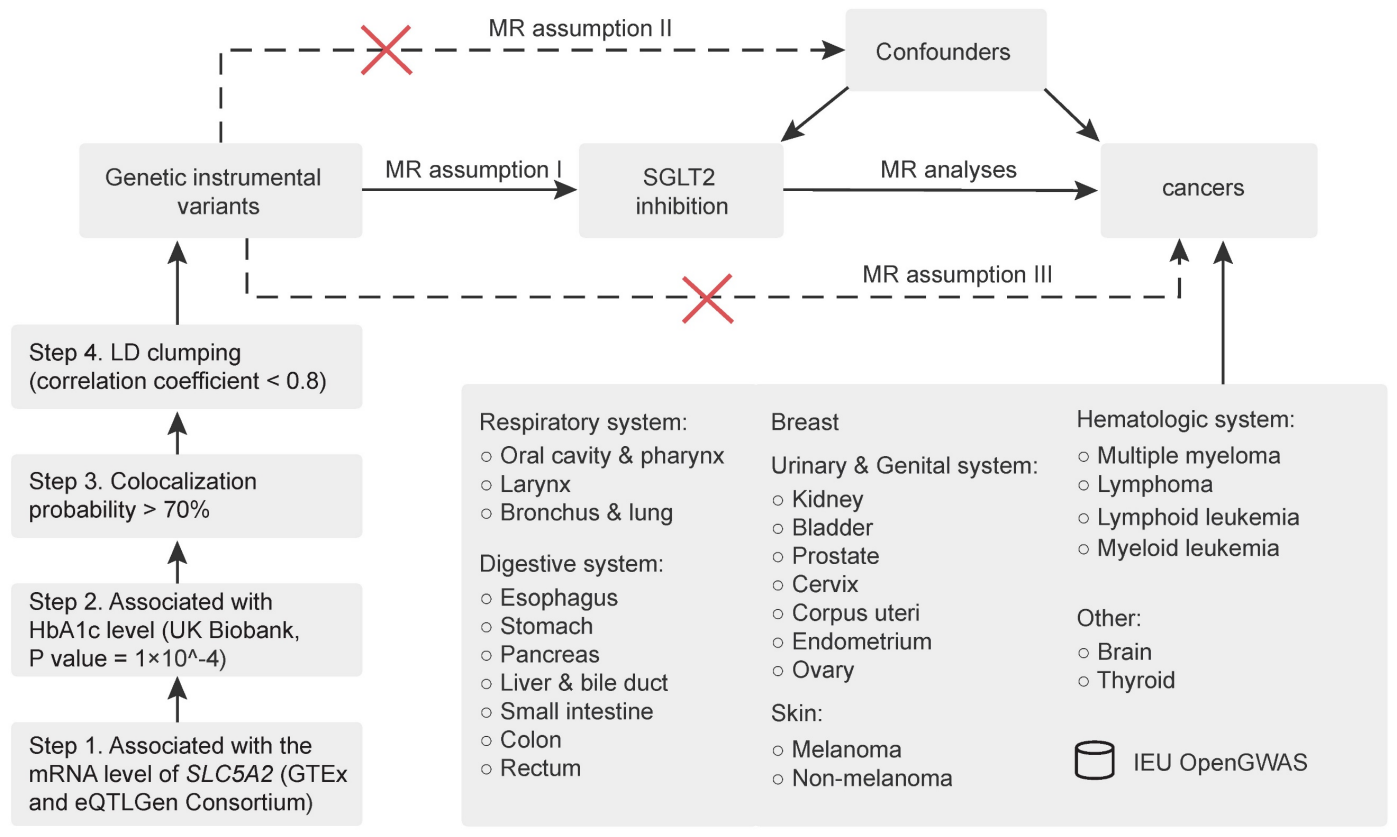
Study design. MR analyses: inverse variance weighted, MR-Egger, weighted median, simple mode and weighted mode. MR assumption I: genetic instrumental variants are strongly associated with the exposure. MR assumption II: genetic instrumental variants are not associated with confounders. MR assumption III: genetic instrumental variants influence outcomes only through the exposure.

The study flowchart is depicted in Figure 1, and the MR study is conducted in compliance with the STROBE-MR statement 20. First, genetic instrumental variables that proxied the effects of SGLT2 inhibition (exposure) were identified. Second, through a thorough review of the published literature concerning the underlying association between SGLT2 and cancer, we finally designated the following sites of malignant neoplasms as the outcome: respiratory system (oral cavity and pharynx, larynx, bronchus and lung), digestive system (esophagus, stomach, pancreas, liver and bile duct, small intestine, colon, and rectum), breast, urinary and genital system (kidney, bladder, prostate, cervix, corpus uteri, endometrium, and ovary), skin (melanoma, non-melanoma), hematologic system (multiple myeloma, lymphoma, lymphoid leukemia, and myeloid leukemia) and others (brain, thyroid). A primarily inverse variance weighted (IVW) method-based two-sample MR analysis was executed to determine the causal relationship between SGLT2 inhibition and cancer risk. Additionally, sensitivity analyses were carried out to further investigate the robustness of the findings, including leave-one-out analysis and tests for heterogeneity and pleiotropy.

For MR assumption I, we evaluated the robustness of genetic instruments using *F* statistics, and we considered it sufficient when *F* statistics exceeded 10. Assumptions Ⅱ and Ⅲ were tested using the PhenoScanner website (http://www.phenoscanner.medschl.cam.ac.uk/) 21 to investigate the association between variants and known risk factors for specific types of cancer, such as body mass index, smoking, alcohol consumption, and lack of physical activity. Variants with a *P*-value lower than 1.0×10^-5^ were excluded from the analysis.

### Selection of genetic instrumental variants for SGLT2 inhibition

A similar genetic instrumental variants selection process was conducted according to Xu *et al.* 22. As depicted in Figure 1, a four-step procedure was adopted: (1) genetic instrumental variants strongly associated with the mRNA level of *SLC5A2* and other potential functional gene of SGLT2 inhibitors were initially selected using genome-wide association studies (GWAS) data from GTEx 23 and eQTLGen Consortium 24, (2) then another reliable marker reflecting the antihyperglycemic effect of SGLT2 inhibitors, glycated hemoglobin A1c (HbA1c), was employed to further screen variants using data from the UK Biobank 25 (https://gwas.mrcieu.ac.uk) (association *P* value = 1 × 10^-4^), (3) causal variants with a probability of genetic colocalization between HbA1c and *SLC5A2* expression of ≤ 0.7 were excluded, and (4) a standard clumping procedure was selected as the final step to remove variants with very high correlation (correlation < 0.8).

### Data sources for cancers

Two main sources of cancer GWAS studies were used to explore the causal relationship between SGLT2 inhibition and cancer risks: (i) discovery stage (Supplementary Table S1): We selected UK Biobank studies as the discovery cohorts because it contains the largest variety of cancer GWAS (cancer cases / controls: oral cavity & pharynx (839/372,016), larynx (273/372,016), bronchus & lung (2,671/372,016), esophagus (740/372,016), stomach (1,029/475,087), pancreas (1,196/475,049), liver & bile duct (350/372,366), small intestine (156/337,003), colon (2,226/358,968), rectum (1,470/461,540), kidney (1,114/461,896), bladder (1,279/372,016), cervix (563/198,523), corpus uteri (1,222/359,972), ovary (1,588/244,932), melanoma (3,751/372,016), non-melanoma (23,694/372,016), multiple myeloma (601/372,016), lymphoma (1,752/359,442), lymphoid leukemia (760/372,016), myeloid leukemia (462/372,016), brain (606/372,016), thyroid (1,054/490,920)). Some GWAS studies were obtained from some large international consortiums or independent team, including the Breast Cancer Association Consortium (BCAC, 14,910 cases and 17,588 controls) 26, the Prostate Cancer Association Group to Investigate Cancer-Associated Alterations in the Genome Consortium (PRACTICAL, 79,194 cases and 61,112 controls) 27, and a genome-wide meta-analysis on endometrial cancer (12,906 cases and 108,979 controls) 28; (ii) replication stage (Supplementary Table S2): GWAS data from the FinnGen database including 26 type of cancers (https://www.finngen.fi) (cancer cases / controls: oral cavity & pharynx (126/218,666), larynx (180/218,612), bronchus & lung (1,681/217,111), stomach (633/218,159), pancreas (605/218,187), liver & bile duct (304/218,488), small intestine (252/218,540), colon (1,803/216,989), rectum (1,078/217,714), breast (8,401/115,178), kidney (971/217,821), bladder (1,115/217,677), prostate (6,311/88,902), cervix (1,648/121,931), corpus uteri (1,053/122,526), endometrium (2,188/237,839), melanoma (393/218,399), non-melanoma (10,382/208,410), multiple myeloma (598/218,194), lymphoid leukemia (663/218,129), myeloid leukemia (283/218,509), brain (464/218,328), thyroid (989/217,803)), esophageal cancer GWAS study was obtained from a large-scale meta-analysis (4,112 cases and 17,159 controls) 29, ovarian cancer GWAS study was obtained from the Ovarian Cancer Association Consortium (OCAC, 25,509 cases and 40,941 controls) 30, endometrial cancer and malignant lymphoma cases were from a meta-analysis 31.

### Statistical analysis

The causal relationship between SGLT2 inhibition and 26 site-specific cancers was estimated using two-sample MR. The main method used for our analysis was the IVW method in order to obtain the maximum statistical power 32. Additionally, we also utilized other MR approaches such as MR-Egger, weighted median, simple mode, and weighted mode for further validation and comparison. We also assessed the robustness and outliers through sensitivity analysis, as well as potential biases (such as genetic pleiotropy and heterogeneity). The presence of horizontal pleiotropy of genetic variants was detected and corrected by the MR Pleiotropy Residual Sum and Outlier (MR-PRESSO) test 33. The heterogeneity estimation was carried out by MR-Egger and IVW methods. *P* > 0.05 was interpreted as indicating no substantial pleiotropy or heterogeneity among the selected genetic variants. In addition, the leave-one-out analysis was applied to ascertain if the causal association between the exposure and the outcome was due to a single genetic variant.

To ascertain the final causative relationship between SGLT2 inhibition and malignancies, a meta-analysis integrating the MR findings from the discovery and replication datasets was carried out. In particular, the MR results were integrated using the fixed-effects model when the heterogeneity was modest (I^2^ < 50%). In cases when there was significant heterogeneity indicated by I^2^ values exceeding 50%, the findings were combined using the random-effects model.

To avoid false positive results, a Bonferroni-corrected *P* value of less than 1.92 × 10^-3^ (0.05/26, for the 26 types of cancer outcomes) was considered significant in the context of the present study, while a *P* value greater than 1.92 × 10^-2^ but less than 0.05 indicated a suggestive causal association. All of the analyses were carried out using the "TwoSampleMR" and "meta" packages in R Studio (version 4.2.1).

## Results

### Characteristics of selected genetic instrumental variants

The characteristics of the six variants (rs8057326, rs11865835, rs34497199, rs4488457, rs35445454 and rs9930811) we used to proxy the pharmacological effects of SGLT2 inhibition were detailed in Supplementary Table S3. Each instrument displayed robust *F* statistics greater than 10, reducing the likelihood of weak instrument bias in this study.

### Discovery results of causal estimation of SGLT2 inhibition on cancer risk

**Figure 2:**
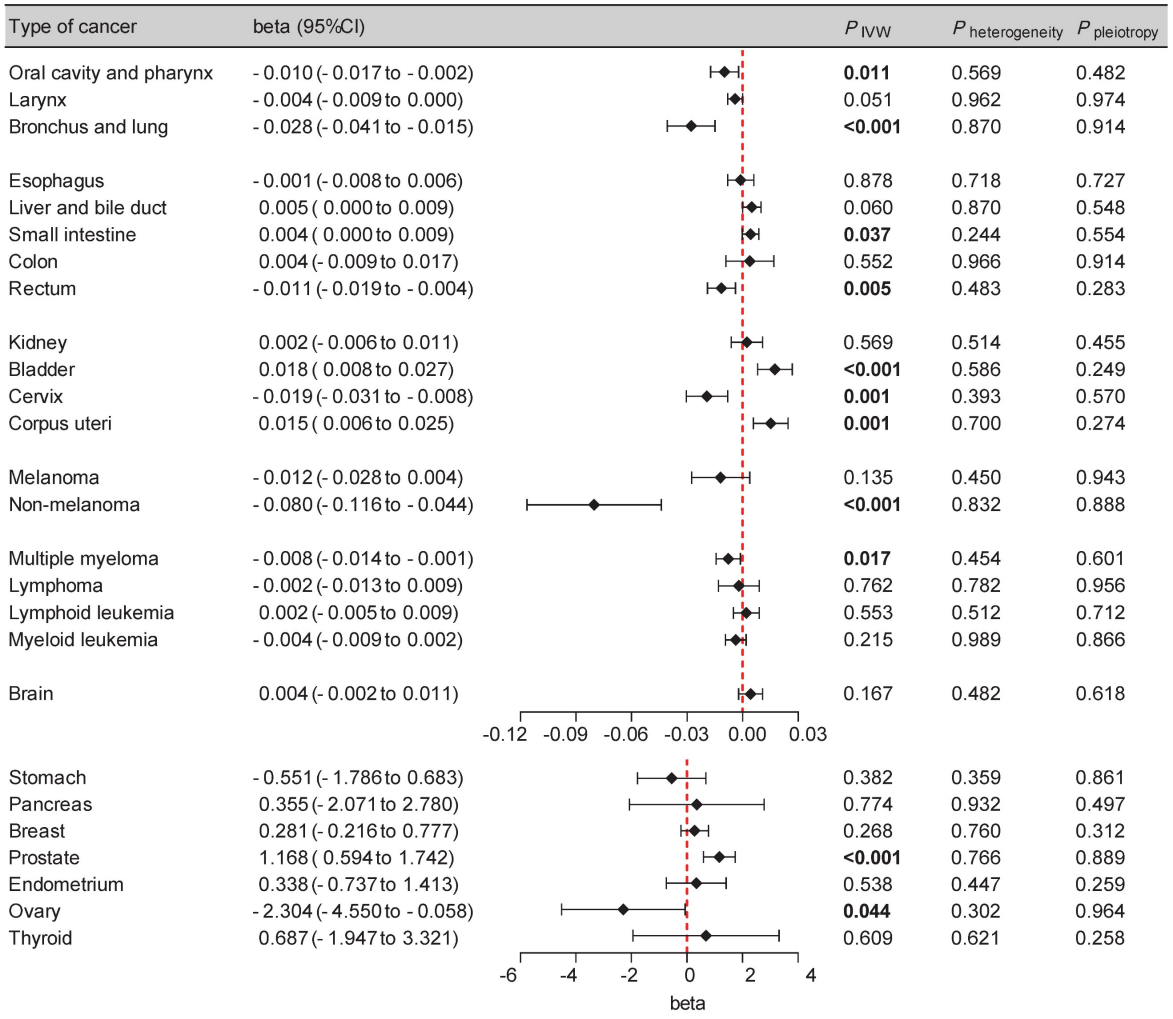
Forest plot of two-sample Mendelian randomization (MR) estimation of the association between SGLT2 inhibition and cancer risk for discovery analysis. CI, confidence interval. IVW, inverse variance weighted.

SGLT2 inhibition was found to be significantly associated with several types of cancers, including bronchial and lung cancer (beta: -0.028 [-0.041, -0.015], *P* < 0.001), bladder cancer (beta: 0.018 [0.008, 0.027], *P* < 0.001), prostate cancer (beta: 1.168 [0.594, 1.742], *P* < 0.001), cervical cancer (beta: -0.019 [-0.031, -0.008], *P* = 0.001), corpus uterine cancer (beta: 0.015 [0.006, 0.025], *P* = 0.001) and non-melanoma skin cancer (beta: -0.080 [-0.116, -0.044], *P* < 0.001). The suggestive causality between SGLT2 inhibition with oral cavity and pharyngeal cancer (beta: -0.010 [-0.017, -0.002], *P* = 0.011), small intestinal cancer (beta: 0.004 [0.000, 0.009], *P* = 0.037), rectal cancer (beta: -0.011 [-0.019, -0.004], *P* = 0.005), ovarian cancer (beta: -2.304 [-4.550, -0.058], *P* = 0.044) and multiple myeloma (beta: -0.008 [-0.014, -0.001], *P* = 0.017) was also noted (Figure 2, Supplementary Table S4). The IVW-based the MR-PRESSO test results implied absence of heterogeneity or horizontal pleiotropy (Figure 2). These results suggest that at the genetic level, SGLT2 inhibition might increase the risk of bladder, prostate, and corpus uterine cancer, while decreasing the risk of other aforementioned cancer types. Furthermore, the leave-one-out analysis supported these findings (Supplementary Table S5).

### Replication results of causal estimation of SGLT2 inhibition on cancer risk

**Figure 3:**
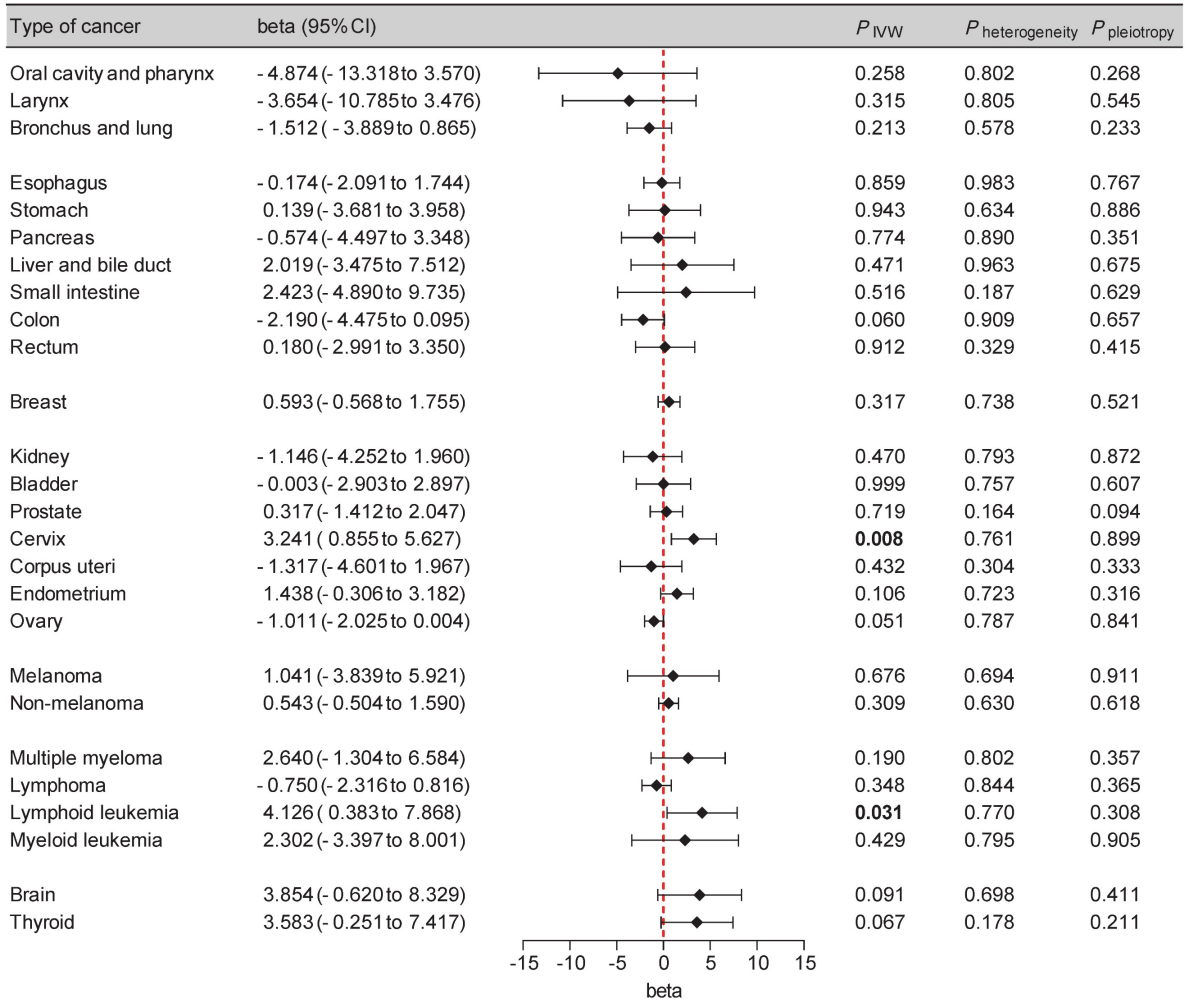
Forest plot of two-sample Mendelian randomization (MR) estimation of the association between SGLT2 inhibition and cancer risk for replication analysis. CI, confidence interval. IVW, inverse variance weighted.

To enhance the reliability of our findings, we incorporated additional cancer GWAS datasets, primarily from the FinnGen database, into our replication analysis (Supplementary Table S2). A potential causal effect between SGLT2 inhibition and an elevated risk of cervical cancer (beta: 3.241 [0.855, 5.627], *P* = 0.008) and lymphoid leukemia (beta: 4.126 [0.383, 7.868], *P* = 0.031) was observed, with non-significant heterogeneity or pleiotropy supporting these findings (Figure 3, Supplementary Table S4). The leave-one-out analysis further supported the robustness of these findings (Supplementary Table S5).

### Final causality of SGLT2 inhibition on cancer risk

MR findings from both the discovery and replication datasets were synthesized using meta-analysis. Detailed results are presented in Table 1. The combined causality of the following cancer types remained significant: bronchial and lung cancer (beta: -0.028 [-0.041, -0.015], *P* < 0.001), bladder cancer (beta: 0.018 [0.008, 0.027], *P* < 0.001), prostate cancer (beta: 1.084 [0.539, 1.628], *P* < 0.001), corpus uterine cancer (beta: 0.015 [0.006, 0.025], *P* = 0.001) and non-melanoma skin cancer (beta: -0.079 [-0.116, -0.043], *P* < 0.001). Additionally, a suggestive association between SGLT2 inhibition and the following malignancies was also confirmed: oral cavity and pharyngeal cancer (beta: -0.010 [-0.017, -0.002], *P* = 0.011), small intestinal cancer (beta: 0.004 [0.000, 0.008], *P* = 0.037), rectal cancer (beta: -0.011 [-0.019, -0.004], *P* = 0.005), ovarian cancer (beta: -1.230 [-2.154, -0.305], *P* = 0.009), and multiple myeloma (beta: -0.008 [-0.014, -0.001], *P* = 0.017).

## Discussion

SGLT2 inhibitors have demonstrated beneficial effects beyond glucose control, predominantly by impeding glucose reabsorption in the proximal renal tubule. As a widely used anti-diabetic drug, it is important to balance the benefits and harms of SGLT2 inhibitors in clinical practice. In addition to cardiorenal protection, SGLT2 inhibitors have been reported to reduce all-cause death and admission to hospital for heart failure, and may improve quality of life 34. Unlike thiazolidinediones, no evidence eliciting fracture risk associated with long-term use of SGLT2 inhibitors 35. Meanwhile, these drugs may also pose the risk of genital infection and ketoacidosis 34. Nevertheless, the influence of SGLT2 inhibitors on cancer remains a contentious issue. Our MR study represents the initial comprehensive examination of the causal association between SGLT2 inhibitors and 26 site-specific malignancies throughout the entire body. Our findings suggested that genetically predicted SGLT2 inhibition played a causal role in reducing the risk of several malignancies, including oropharyngeal cancer, bronchial and lung cancer, rectal cancer, ovarian cancer, non-melanoma skin cancer, and multiple myeloma. Conversely, it seemed to elevate the likelihood of developing cancers of the small intestine, bladder, prostate, and corpus uteri. These insights may shed light on the efficacy of this commonly prescribed medication.

Hyperglycemia is widely believed to augment cancer risk and diminish the response to chemotherapy by directly affecting cell proliferation and drug resistance 36-38. Generally, individuals with diabetes were found to face an elevated risk of developing several types of cancer, including digestive system tumors, breast and endometrial (in women), and kidney 39. Since the advent of SGLT2 inhibitors, an increasing body of evidence has supported their potential application in cancer treatment. Mechanically, SGLT2 inhibitors may exert beneficial effects on cancer initiation and progression through the regulation of crucial cancer hallmarks such as cellular growth, oxidative stress, and inflammatory responses. This is accomplished by altering distinct molecular pathways within various cancer types 40-43.

However, clinical trials investigating the impact of SGTL2 inhibitors on cancer risk have yielded conflicting results. Preclinical trials involving the SGLT2 inhibitor dapagliflozin indicated an elevated risk of bladder cancer and breast cancer 44. A meta-analysis conducted by Tang and colleagues suggested an increased risk of bladder cancer, particularly with empagliflozin, while canagliflozin appeared to offer protection against gastrointestinal cancers 8. Yet, these findings were less than conclusive due to the short duration of the trials and the uncertainty of the data. In contrast, an international multisite cohort study offered reassurance regarding the short-term efficacy of SGLT2 inhibitors, uncovering no elevated hazard of bladder cancer in comparison to glucagon-like peptide 1 receptor agonists (GLP1-RAs) or dipeptidyl peptidase IV inhibitors (DPP-4i) 45. Another meta-analysis reported a significant overall reduction in cancer risk associated with SGLT2 inhibitors compared to placebo (relative risk [RR]: 0.35 [0.33, 0.37]), with dapagliflozin (RR: 0. 06 [0. 06, 0. 07]) and ertugliflozin (RR: 0. 22 [0. 18, 0. 26]) showing particular effectiveness 17.

In our study, we discovered a causal association between SGLT2 inhibition and a 1.8% increased risk of bladder cancer, a potential 0.4% elevated risk of small intestinal cancer, and a potential 1.1% reduced risk of rectal cancer. A retrospective cohort study involving adult patients with T2DM and colorectal adenocarcinoma suggested that SGLT2 inhibitor recipients had improved overall survival rates compared to non-recipients 46. Concurrently, SGLT2 inhibitors were linked to a 50-70% reduction in all-cause mortality and disease progression. Another territory-wide cohort study found a significant association between SGLT2 inhibitor use and decreased incidence of colorectal cancer in younger patients, men and patients with preserved renal function 47. Our findings lend further support to the protective effect of SGLT2 inhibitors on rectal cancer from a genetic standpoint. With respect to small intestinal cancer, we hypothesized that the potential effect of SGLT2 inhibition might be partially related to SGLT1, an isoform of SGLT2 present in the intestine, liver, lung, brain, and salivary glands 48. Remarkably, some gliflozins exert a dual effect on both SGLT1 and SGLT2 49. However, we found no relevant epidemiological studies on SGLT2 inhibition and small intestinal cancer, and the effect size was extremely close to 1, suggesting that this finding should be interpreted cautiously.

Our MR study also found a causal association between SGLT2 inhibition and a 2.8% decreased risk of bronchus and lung cancer. A study on the impact of SGLT2 inhibitors on survival in patients with non-small cell lung cancer (NSCLC) revealed that the use of these inhibitors was linked to prolonged overall survival in NSCLC patients who had diabetes prior to diagnosis, irrespective of demographic factors, tumor features, and treatment modalities 50. An *in vitro* study using a lung model found that canagliflozin had an anticancer effect, inhibiting the proliferation of A549 lung cancer cells by blocking cell cycle progression 51. Furthermore, SGLT2 has been identified as a potential marker for early-stage lung adenocarcinoma (LADC). Specifically, gliflozins that are selectively targeted at SGLT2 showed promising results in reducing tumor growth and improving survival rates in both murine models and patient-derived xenografts of LADC 12. We also noted a 195.6% increased risk of prostate cancer in our study, which aligns with another MR study 19. Upon reviewing the literature, we found that current evidence regarding the impact of SGLT2 inhibition on prostate cancer remains limited. A study examining the effect of empagliflozin on the urothelium of diabetic and non-diabetic animals observed abnormal dysplastic urothelial changes, such as increased proliferative activity 52.

Additionally, we observed a 1.5% increased risk of malignant neoplasm of the corpus uteri. There is limited evidence directly establishing an association between uterine cancer and SGLT2 inhibitors use. A pertinent study investigating the anticancer effect of empagliflozin on cervical carcinoma models demonstrated that empagliflozin could regulate the expression of Sonic Hedgehog Signaling Molecule, thereby inhibiting cell migration and inducing cell death in cervical cancer cells 53. However, we considered the association between SGLT2 inhibition and malignant neoplasm of corpus uteri was not robust enough to infer a causal relationship, given the odds ratio value close to 1.

As previously reported, a discrepancy was observed in the number of malignant melanoma cases associated with empagliflozin use 54. However, a combined analysis failed to establish a direct link between SGLT2 inhibitors and the overall likelihood of developing skin cancer in individuals with T2DM 55. Specifically, there was a slight rise in the risk of melanoma among those using SGLT2 inhibitors, although it was not statistically significant (odds ratio [OR]: 2.17 [0.80, 5.89]). Nevertheless, when examining the risk of non-melanoma skin cancer, a notable decrease in risk was observed in studies lasting less than 52 weeks (OR: 0.12 [0.02, 0.59]). It is worth noting that the author suggested that this significance might be attributed to a limited number of occurrences, or a difference in the frequency of pre-existing non-melanoma skin cancer cases between groups at baseline. Similarly, the present study also failed to establish a causal association between SGLT2 inhibition and melanoma. Nevertheless, we pointed out direct causality between SGLT2 inhibition and a 7.6% lower risk of non-melanoma. Therefore, our findings suggested that SGLT2 inhibitors may confer a protective effect on non-melanoma skin cancer from a genetic perspective, necessitating further evidence from future research.

Finally, we also observed a suggestive association between SGLT2 inhibition and reduced risk of multiple myeloma. Nakachi *et al.* demonstrated that blocking SGLT2 can effectively restrain the growth of adult T-cell leukemia cells by inhibiting glucose uptake, leading to decreased intracellular levels of ATP and NADPH. This may exacerbate cell cycle arrest 56. Unfortunately, we were unable to identify the causal effect of genetically proxied SGLT2 inhibition on leukemia. Although licensed for the treatment of relapsed/refractory multiple myeloma, carfilzomib (CFZ) has limited clinical utility due to its cardiovascular toxicity. A recent study has shown that canagliflozin could mitigate the apoptotic impact of CFZ on endothelial while maintaining its anticancer efficacy 57. Rokszin *et al.* demonstrated a reduced risk of hematological malignancies (hazard ratio: 0.50 [0.28, 0.88]) in patients treated with SGLT2 inhibitors compared to those on DPP-4i 58.

Currently, the majority of researches exploring the relationship between SGLT2 inhibitors and malignancies were derived from clinical trials investigating the short-term effects of SGLT2 inhibitors, such as their cardiovascular protective effects. However, long-term and large-scale clinical trials are required to analyze the potential carcinogenic or anticancer effects of these drugs. Consequently, we cannot definitively establish the causal relationship between SGLT2 inhibitors and cancer development based on the currently available clinical data. The MR study is less susceptible to confounding factors and can directly assess the causal effect of SGLT2 inhibition on cancer risk, unlike observational studies, which may be prone to potential confounders. However, it is undeniable that MR method also has inherent limitations. The assumption that the employed genetic variants exclusively influence the outcome via the exposure of interest, represents a fundamental limitation of MR analysis. In this study, we have made rigorous efforts to ascertain the robustness of MR results. Initially, we searched the PhenoScanner website to eliminate potential confounding factors. Subsequently, sensitivity analyses were conducted to exclude outliers among variants and confirm the absence of pleiotropy. We believe that these procedures significantly reduce bias and enhance the credibility of our results.

Our study has several advantages. First, we used meta-analysis to integrate cancer data from different large databases to summarize the effects of SGLT2 inhibitors on cancer, which avoided selection bias to a certain extent and increased the credibility of our results. Second, this is the first MR study to reveal a possible association between SGLT2 inhibitors and lung cancer, non-melanoma skin cancer, and uterine cancer, which may shed light on the unexplored efficacy of this commonly prescribed medication. We acknowledge that our study still has several limitations. First, the limited cases of certain types of cancer in the UK Biobank or FinnGen database might reduce our statistical power, potentially making them unrepresentative. Second, although we found a significant link between SGLT2 inhibition and malignant neoplasms of the bronchus and lung, bladder, corpus uteri, and non-melanoma, the observed OR values were relatively small, indicating a less robust association. Furthermore, given that this study was data-driven, the lack of external real-world data to support the causal relationship between SGLT2 and cancers was one of the limitations that cannot be ignored. Third, since the study population in this research was of European heritage, our findings may not be generalizable to other ethnicities. These results should be validated in a more diverse range of ethnicities. Furthermore, the potential impact of SGLT2 inhibition on cancer necessitates further investigation through experimental and clinical trials.

## Conclusions

In summary, our present study represents the initial investigation into the causative relationships between SGLT2 inhibition and the risk of 26 site-specific malignancies throughout the body. This comprehensive MR study suggested that SGLT2 inhibition may influence the cancer risk of bronchial and lung, non-melanoma, bladder, prostate, and corpus uteri, which warrants real-world data validation in the future.

## Supplementary Material

Supplementary tables.

## Figures and Tables

**Table 1:** Combined results of SGLT2 inhibitors on 26 site-specific cancers in the discovery and replication genome-wide association studies.

Outcomes	Common effect model		Random effect model		Heterogeneity			
	beta (95% CI)	*P*	beta (95% CI)	*P*	I^2^ (%)	Q	df	*P*
Oral cavity & pharynx	-0.010 (-0.017, -0.002)	**.011**	-0.534 (-3.474, 2.423)	.723	21.6	1.27	1	.259
Larynx	-0.004 (-0.009, 0.000)	.051	-0.016 (-0.420, 0.389)	.938	0.6	1.01	1	.316
Bronchus & lung	-0.028 (-0.041, -0.015)	**<.001**	-0.274 (-1.355, 0.808)	.619	33.2	1.50	1	.221
Esophagus	-0.001 (-0.008, 0.006)	.877	-0.001 (-0.007, 0.007)	.877	0	0.03	1	.860
Stomach	-0.486 (-1.661, 0.689)	.418	-0.486 (-1.661, 0.689)	.418	0	0.11	1	.736
Pancreas	0.098 (-1.965, 2.161)	.926	0.098 (-1.966, 2.161)	.926	0	0.16	1	.693
Liver & bile duct	0.005 (0.000, 0.009)	.060	0.005 (0.000, 0.010)	.060	0	0.52	1	.472
Small intestine	0.004 (0.000, 0.008)	**.037**	0.004 (0.000, 0.009)	.037	0	0.42	1	.517
Colon	-0.784 (-2.846, 1.279)	.457	-0.783 (-2.847, 1.280)	.457	71.8	3.54	1	.060
Rectum	-0.011 (-0.019, -0.004)	**.005**	-0.011 (-0.019, -0.003)	.005	0	0.01	1	.906
Breast	0.329 (-0.128, 0.786)	.158	0.329 (-0.128, 0.786)	.158	0	0.24	1	.628
Kidney	0.002 (-0.006, 0.011)	.570	0.002 (-0.006, 0.011)	.570	0	0.52	1	.469
Bladder	0.018 (0.008, 0.027)	**<.001**	0.018 (0.008, 0.027)	<.001	0	0.00	1	.989
Prostate	1.084 (0.539, 1.628)	**<.001**	1.084 (0.539, 1.628)	<.001	0	0.84	1	.360
Cervix	1.384 (-1.780, 4.547)	.391	1.384 (-1.778, 4.547)	.391	86.1	7.17	1	.007
Corpus uteri	0.015 (0.006, 0.025)	**.001**	0.015 (0.006, 0.025)	.001	0	0.63	1	.427
Endometrium	0.641 (-0.274, 1.556)	.170	0.665 (-0.320, 1.650)	.186	9.7	1.11	1	.293
Ovary	-1.230 (-2.154, -0.305)	**.009**	-1.252 (-2.244, -0.264)	.013	5.5	1.06	1	.304
Melanoma	-0.012 (-0.027, 0.004)	.135	-0.012 (-0.027, 0.004)	.135	0	0.18	1	.672
Non-melanoma	-0.079 (-0.116, -0.043)	**<.001**	0.003 (-0.412, 0.417)	.990	26.4	1.36	1	.244
Multiple myeloma	-0.008 (-0.014, -0.001)	**.017**	0.551 (-1.565, 2.669)	.610	42.2	1.73	1	.188
Lymphoma	-0.002 (-0.013, 0.009)	.757	-0.002 (-0.013, 0.009)	.757	0	0.88	1	.349
Lymphoid leukemia	1.622 (-2.325, 5.569)	.421	1.622 (-2.323, 5.569)	.421	78.6	4.66	1	.031
Myeloid leukemia	-0.004 (-0.009, 0.002)	.216	-0.003 (-0.009, 0.002)	.216	0	0.63	1	.428
Brain	1.253 (-2.280, 4.785)	.487	1.253 (-2.283, 4.785)	.487	64.8	2.84	1	.092
Thyroid	1.616 (-0.555, 3.787)	.145	1.786 (-0.968, 4.540)	.204	32.8	1.49	1	.223

Abbreviations: CI, confidence interval.

## Abbreviations

SGLT2: sodium-glucose cotransporter 2
T2DM: type 2 diabetes mellitus
MR: mendelian randomization
IVW: inverse variance weighted
GWAS: genome-wide association studies
HbA1c: glycated hemoglobin A1c
MR-PRESSO: mendelian randomization pleiotropy residual sum and outlier
GLP1-RAs: glucagon-like peptide 1 receptor agonists
DPP-4i: dipeptidyl peptidase IV inhibitors
RR: relative risk
SGLT1: sodium-glucose cotransporter 1
NSCLC: non-small cell lung cancer
LADC: lung adenocarcinoma
OR: odds ratio
CFZ: carfilzomib
